# Newcastle disease virus hijacks chicken red blood cells as dissemination vehicles with concomitant adsorption-mediated cell death

**DOI:** 10.1186/s13567-025-01684-9

**Published:** 2025-12-16

**Authors:** Qingyi Wang, Yang Qu, Yingjie Sun, Ying Liao, Xusheng Qiu, Lei Tan, Cuiping Song, Ning Tang, Libin Chen, Tao Ren, Chan Ding

**Affiliations:** 1 College of Veterinary Medicine, South China Agricultural University, Guangzhou, China; 2 Department of Avian Infectious Diseases, Shanghai Veterinary Research Institute, Chinese Academy of Agricultural Science, Shanghai, China; 3 School of Agriculture and Biology, Shanghai Jiao Tong University, Shanghai, China

**Keywords:** Newcastle disease virus, red blood cells, apoptosis, pathogenic mechanism

## Abstract

Red blood cells (RBCs) are the most abundant cell type in the blood and play a critical role as the primary carriers of oxygen to tissues and organs through blood circulation. The hemagglutinin-neuraminidase (HN) protein on the surface of the Newcastle disease virus (NDV) contains receptors that bind to the surface of RBCs, endowing NDV with agglutination properties that hold significant clinical diagnostic value. This raises an important question: could NDV bind to RBCs and use their carrier properties to facilitate transport to various tissues and organs? This study conducted both in vivo and in vitro experiments to confirm the adhesion and transport capabilities of chicken RBCs for the Newcastle disease virus. In addition, we found that NDV infection induces apoptosis in RBCs. These findings systematically explored the infection process of NDV in chicken RBCs and its subsequent effects, providing direct evidence of the potential role of chicken RBCs as a transport vehicle for the virus. This research offers a novel perspective on the mechanisms of NDV transmission.

## Introduction

Newcastle disease (ND) is an acute and highly contagious infectious disease caused by virulent strains of NDV. It affects over 250 species of wild birds and poultry [1, 2]. This disease poses a significant challenge to the poultry industry and presents serious risks to food security and public health [3, 4].

The primary transmission routes for ND are through the digestive and respiratory tracts. The virus initially replicates in the respiratory tract and intestines, before rapidly entering the bloodstream and spreading systemically. Once in the bloodstream, the virus causes extensive damage to the vascular walls, leading to severe hemorrhage, serous exudation, and tissue necrosis [5].

These effects are often accompanied by gastrointestinal disturbances, pulmonary congestion, and central respiratory dysfunction due to circulatory disorders. In the late stages of some chronic cases, the viral load in the blood may significantly decrease. Additionally, certain strains can severely damage lymphoid tissues such as the thymus and spleen, resulting in immunosuppression [6].

In summary, the circulatory system plays a critical role in the systemic spread of NDV, contributing to widespread tissue damage and various clinical manifestations of the disease.

RBCs are not only the most abundant cells in the blood but are also among the most prevalent cell types in the entire organism [7]. Their primary function is to transport oxygen and carbon dioxide throughout the bloodstream [8]. In recent years, the role of RBCs in the immune system has received increasing attention [9–11]. Research has shown that RBCs can directly bind to and eliminate antigens in the bloodstream, challenging the traditional view that only white blood cells are involved in cellular immunity. This discovery opens new avenues for investigating the immune functions of red blood cells [12].

In addition, there are reports indicating that RBCs produce specific immune molecules that interact with pathogens, forming immune complexes. These immune complexes are then cleared from the bloodstream by being presented to immune cells or phagocytes for further processing [13–15]. Moreover, when stimulated by pathogen-associated molecular patterns, RBCs initiate the transcription of various immune response factors, including interferons and their downstream interferon-stimulated genes [9].

In summary, these findings suggest that, beyond their role in carrier transport, RBCs act as an initial line of defence against pathogenic microorganisms in the body. Therefore, it is essential to further investigate the interaction between viral infections and RBCs.

A range of receptor proteins present on the surface of RBCs play a crucial role in facilitating virus adsorption and infection [16–18]. The hemagglutinin-neuraminic (HN) protein on the surface of NDV specifically binds to these receptors, demonstrating strong red blood cell agglutination properties. However, the interaction between the virus and RBCs is not permanent [19]. When the neuraminidase on the surface of the virus and the RBC receptors become compromised or damaged, the virus eventually dissociates from the RBCs. This process allows NDV to use RBCs as vectors for transport within the host.

While the regulatory mechanisms of mammalian erythropoiesis have been extensively studied, research on cell death in RBCs has largely concentrated on “eryptosis” [20, 21]. Notably, there are no reported incidents of avian RBC death induced by viral infection. Similar to other paramyxoviruses, NDV is known to induce hemolysis of RBCs, resulting in the release of a significant amount of hemoglobin. This process is primarily attributed to the fusion of the viral envelope with the red blood cell membrane [22, 23]. However, it is still unclear whether this process is accompanied by the externalization of phosphatidylserine (PS) on the surface of red blood cells, which is a hallmark of apoptosis [24], and whether it triggers programmed cell death in these cells.

In this study, we investigated the damage and death of chicken RBCs caused by NDV infection both in vivo and in vitro. We focused on the replication and proliferation of NDV within infected RBCs and highlighted the role of RBCs as carriers for the virus. Our findings confirm that RBCs can serve as carriers for NDV, contributing to systemic infection. By examining the infection process of NDV in chicken RBCs and its effects, we have addressed a research gap concerning NDV infection in these cells, thus providing a theoretical foundation for understanding the pathogenic mechanisms associated with NDV infection.

## Materials and methods

### Cells, viruses, and animals

DF-1 cell lines were obtained from the American Type Culture Collection (ATCC). CEFs were prepared from 10 day-old embryonated eggs as previously described [25]. The NDV Herts/33 strain was sourced from the China Institute of Veterinary Drug Control (Beijing, China). The NDV RFP-LaSota strains were generated and stored in our laboratory [26]. The virus strain was multiplied in 9-day-old embryonated specific-pathogen-free (SPF) chicken eggs and stored at −80 °C. The determination of the 50% tissue culture infective dose (TCID_50_) was performed as described previously [27]. All SPF embryonated eggs were purchased from Beijing Boehringer Ingelheim Vital Biotechnology Co., Ltd. (Beijing, China) and incubated as previously described [28, 29].

### Animal challenge experiments

The hatched SPF chickens were housed in a sterile, negative-pressure isolator until they reached 3 weeks of age. During this period, they had continuous access to drinking water and food. The test group was inoculated via the jugular vein with 0.2 mL of NDV Herts33 strain allantoic fluid [10^7^ plaque-forming units (PFU)] per animal, while the control group received an equivalent volume of PBS buffer.

At designated time points post-challenge, three animals were randomly selected for heart blood collection, and RBCs were isolated for further experimentation. Bleeding was performed under anesthesia using ketamine and xylazine. All the challenged animals were euthanized with carbon dioxide when they became moribund.

### Chicken RBCs preparation and viral infection

The hatched SPF chickens were housed in a sterile, negative-pressure isolator until they reached 3 weeks of age. They had continuous access to drinking water and food. Blood was aseptically collected from the subwing vein into anticoagulant tubes and mixed with an equal volume of Albright’s solution (Solarbio, Beijing, China). The blood was then carefully added to 4 mL of Histopaque-1119 solution (Sigma–Aldrich, Oakville, ON) and centrifuged at 1000 rpm for 20 min. After centrifugation, the supernatant containing platelets and leukocytes was removed [30].

RBCs (10^6^ cells) were cultured in Dulbecco’s modified eagle medium (DMEM) supplemented with 10% fetal bovine serum (FBS). The cells were directly mixed with NDV and incubated in a 37 °C incubator for 2 h. Following incubation, the mixture was centrifuged at 1000 rpm for 5 min, and the RBCs were recultured in DMEM for a specified duration before further analysis. This setup was referred to as the preincubation group.

Afterwards, the RBCs and NDV were coincubated in a 37 °C incubator for a designated period, centrifuged at 1000 rpm for 5 min, and the RBCs were pelleted and used directly for subsequent analysis. This setup was defined as the coincubated group.

### Measurement of hemoglobin

The Hemoglobin Colorimetric Assay Kit (P0381S; Beyotime) was used to measure the hemoglobin concentration in both serum and red blood cell culture supernatants. For serum sample analysis, whole blood was collected and allowed to coagulate naturally at room temperature for 30 min. The sample was then centrifuged at 3000 rpm for 5 min, and the yellowish serum layer at the top was carefully collected for detection.

For the analysis of cultured RBCs in vitro, the RBCs incubated with viruses were centrifuged at 1000 rpm for 5 min, and the supernatant was collected for further testing. Following the manufacturer’s instructions, a luminometer was used to measure the luminescence rate of the standard and samples. The standard curve was then created to calculate the concentration of hemoglobin in the samples.

### Quantitative real-time PCR

Total RNA was extracted from NDV-infected RBCs or tissue samples using the Cell/Tissue Total RNA Kit (19221ES50, YEASEN). The extracted RNA was then reverse-transcribed into cDNA using the One-Step gDNA Removal and cDNA Synthesis Super Mix (AE311; TransGen Biotech, Beijing, China) following the manufacturer’s instructions. Quantitative real-time PCR was performed using the Universal Blue qPCR SYBR Green Master Mix (11184ES08, YEASEN) and the following primers: NDV-NP, 5′-CAACAATAGGAGTGGAGTGTCTGA-3′ (F) and 5′-CAGGGTATCGGTGATGTCTTCT-3′ (R); chicken *β*-actin, 5′-AGACATCAGGGTGTGATGG-3′ (F) and 5′-TCAGGGGCTACTCTCAGCTC-3′ (R); chicken Bcl-2, 5′-GGATCGTCGCCTTCTTCGAG-3′ (F) and 5′-CCACAAAGGCATCCCATCCTC-3′ (R); chicken MCL-1, 5′-GGACGCATTGGTCTCATCCA-3′ (F) and 5′-ACTCCTCCACTTTCGGATCA -3′(R); chicken Bak, 5′-CCCTATTCGCTTCCTTCCCC-3′ (F) and 5′-GGACTCAGCAGGACCGTG-3′ (R); chicken MLKL, 5′-TTACATTGCCCCCGAGAACC-3′ and 5′-GGAGAGGGCAGCCTTCAAAT-3′ (R); chicken PARP, 5′-GATCCTGATTCCGGCTTGGA-3′ and 5′-CGACCCCAGGATCTGAACAC-3′. All experiments were carried out in triplicate.

### Virus adsorption and internalization experiments

In the adsorption experiment, fresh RBCs were precooled at 4 °C for 15 min and then incubated with NDV at an multiplicity of infection (MOI) of 5 for 2 h at 4 °C. After the incubation, the supernatant was discarded, and the cells were washed three times with PBS to remove any unadsorbed virion particles.

For the internalization experiment, the virus was first incubated with RBCs at 4 °C for 2 h, following the same procedure as the adsorption experiment. Next, the cells were shifted to 37 °C and incubated for 2 h to allow for viral internalization. After this incubation, the cells were treated with citrate buffer (50 mM sodium citrate, 4 mM potassium chloride, pH 3.0) to remove any noninternalized virion particles [31].

Finally, the cells were lysed to extract total RNA, and the viral genome copy number was analyzed using RT-qPCR.

### Detection of red blood cell survival rate

Fresh RBCs were collected and diluted to a 10% cell suspension using PBS buffer. The survival rate of the RBCs was then evaluated using an automatic cell counting and viability analyzer (Countstar, Shanghai, China).

### Blood smear preparation

A total of 5 μL of fresh anticoagulated blood was placed at the one-quarter mark of a clean glass slide. The smooth edge of the spreader was positioned at a 45° angle in front of the blood drop and gently pulled back to contact the blood drop, allowing the blood to spread evenly along the lower edge of the spreader in a straight line. The spreader was pushed forward to the other end of the slide. The smear was immediately placed horizontally and allowed to air dry naturally. Once the smear was completely dry, Swiss staining was performed according to the instructions (C0135; Beyotime). After the smear was fully stained and dried, we observed the morphology of the RBCs under a microscope.

### RNA sequencing

Fresh RBCs were collected according to the design of the animal challenge experiments. They were immediately snap-frozen in liquid nitrogen and stored at −80 °C until further processing. Total RNA was extracted from the samples, and its concentration, purity, and integrity were evaluated. The samples were then sent to the Shanghai Majorbio Institute for sequencing, and the resulting data were analyzed using the online Majorbio Cloud Platform. In addition, a Kyoto encyclopedia of genes and genomes (KEGG) pathway enrichment analysis was conducted, following the methods described in previous studies [32].

### Flow cytometry analysis of apoptosis

Fresh RBCs were incubated with NDV for 18 h. Apoptosis was detected using the Apoptosis Detection Kit (C1062L, Beyotime, Shanghai, China) according to the manufacturer’s instructions. Specifically, the RBCs were collected and washed twice with PBS, then resuspended in 195 μL Annexin V-FITC binding buffer. They were incubated with Annexin V and PI at 25 °C for 20 min in the dark. The resulting preparations were analyzed using a flow cytometer (Beckman, California, USA) equipped with FlowJo software.

### Statistical analysis

All data were analyzed using GraphPad Prism version 9.0 software (GraphPad Software, Inc.) and are expressed as means ± standard deviations from at least three independent experiments. Statistical significance was analyzed using two-tailed independent Student’s *t*-tests between the two groups. *P*-values < 0.05 were considered statistically significant.

## Results

### NDV-induced damage in chicken RBCs

Infection with NDV leads to systemic pinpoint hemorrhages in the mucous and serous membranes, such as those in the intestines and glandular stomach. This occurs primarily due to blood extravasation caused by damage to endothelial cells. Once RBCs leak into the tissues, they may experience secondary rupture, potentially triggered by oxidative stress or mechanical damage [6]. However, it is still uncertain whether NDV directly damages the RBCs.

**Figure 1:**
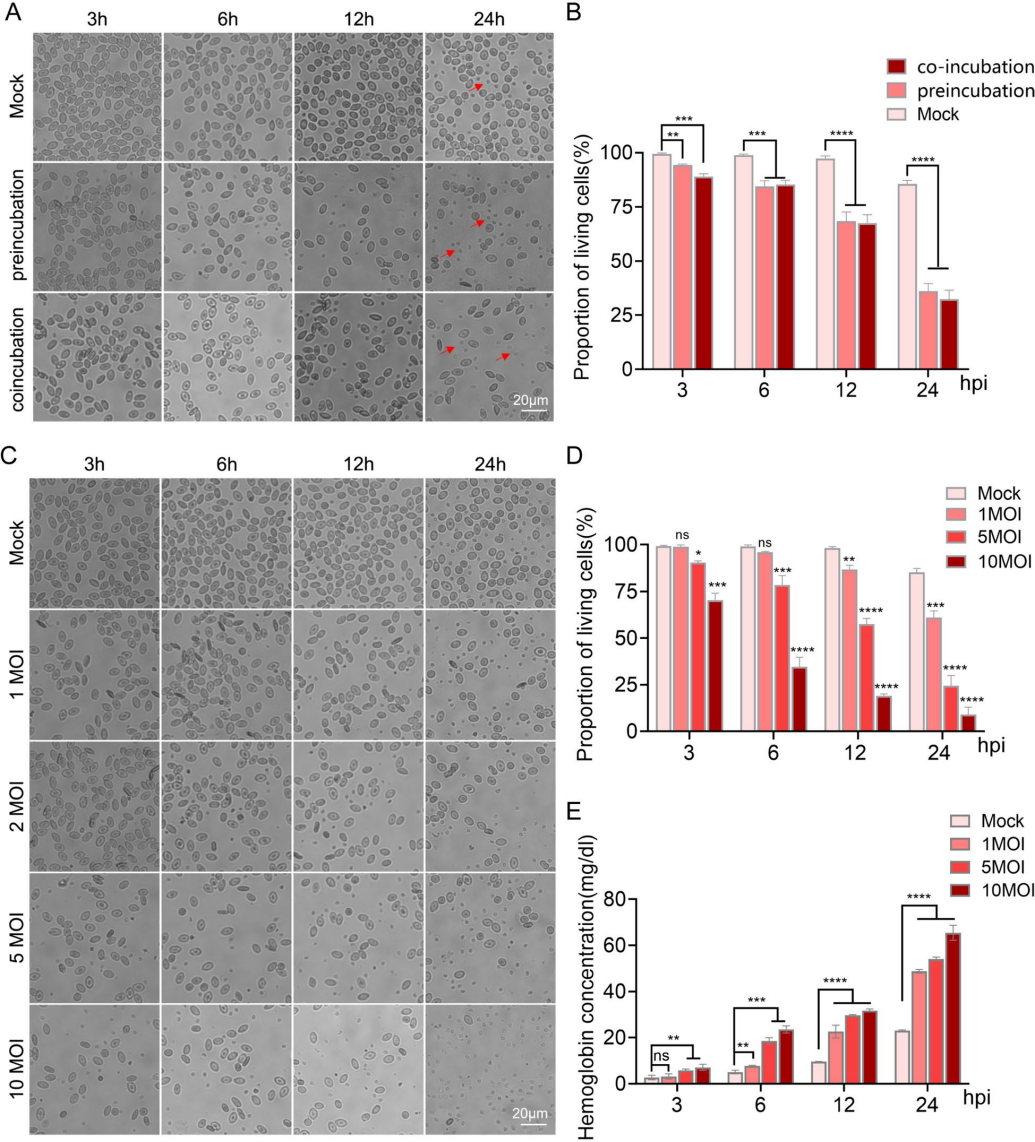
**Newcastle disease virus (NDV)-induced damage in chicken red blood cells (RBCs)**. **A** and **B** Fresh chicken RBCs were infected with NDV through both preincubation and coincubation methods, at 3, 6, 12, and 24 h post-infection (hpi), (**A**) the morphology of the RBCs were observed using a microscope; (**B**) quantification of the percentage of viable cells. **C**–**E** NDV was introduced to fresh RBCs at multiplicities of infection (MOI) of 1, 2, 5, and 10. At the same time intervals (3, 6, 12, and 24 h), (**C**) microscopic observation of the chicken red blood cells; (**D**) quantification of the percentage of viable cells; and (**E**) collection of the cell culture supernatant for the detection of free hemoglobin content. Each bar represents the mean ± standard deviation; *P < 0.05, **P < 0.01, ***P < 0.001 ****P < 0.0001, and ns, not significant.

To investigate this, we isolated fresh chicken RBCs and developed an in vitro NDV infection model. The results indicated that, as the duration of infection increased, the number of damaged RBCs gradually rose (Figure 1A), while cell viability decreased (Figure 1B). To better simulate in vivo infection, we coincubated NDV with RBCs, and the results were consistent with our initial findings (Figures 1A and B).

Subsequent dose–response experiments demonstrated that higher viral doses resulted in more severe cell damage and a further decrease in cell survival rates (Figures 1C, D). Furthermore, the hemoglobin content in the cell culture supernatant increased owing to the damage to red blood cells (Figure 1E). These findings confirm that NDV can directly damage RBCs, leading to the release of hemoglobin.

### NDV did not replicate in chicken RBCs

**Figure 2:**
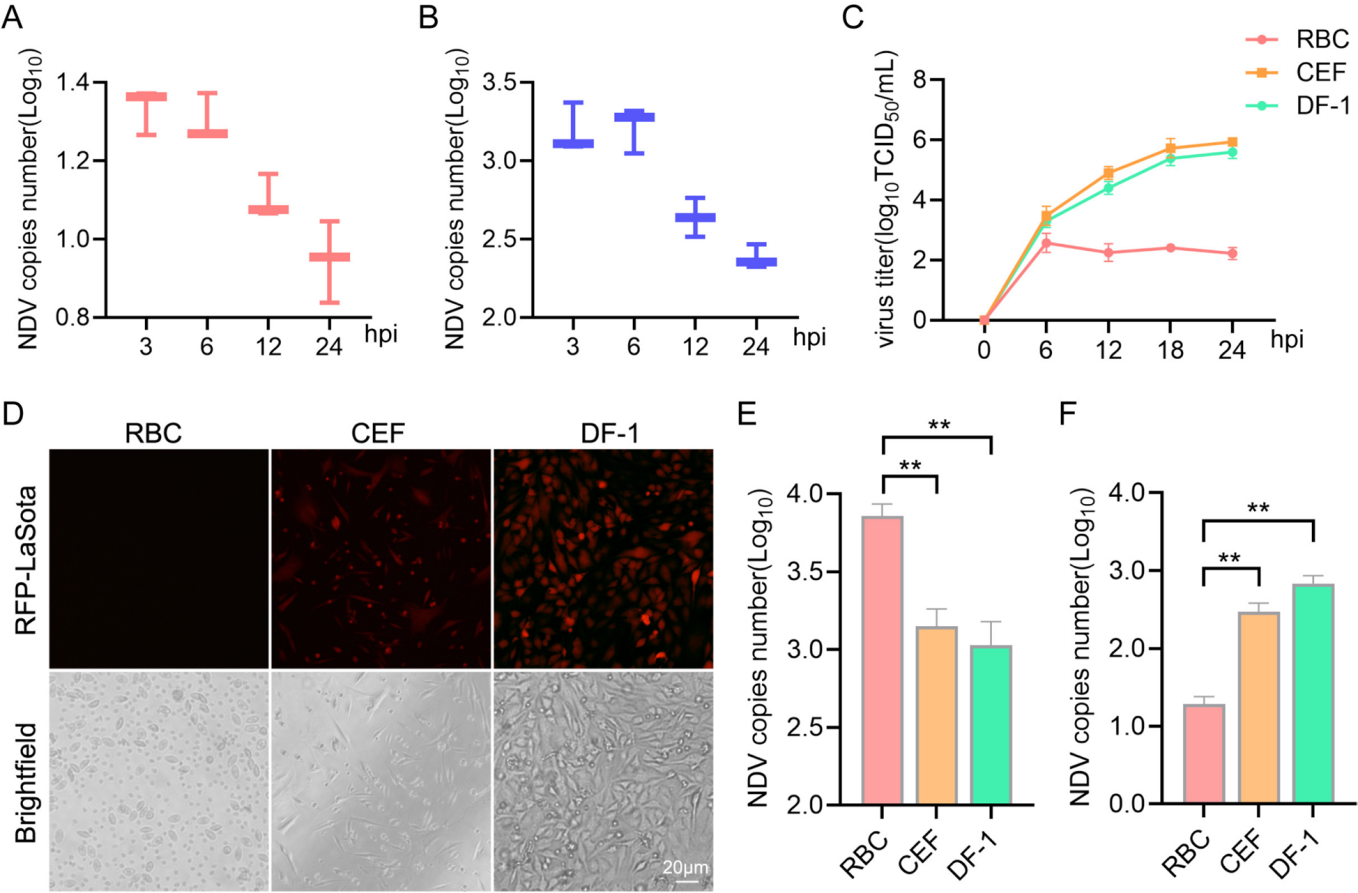
**NDV does not replicate in chicken RBCs**. **A** and **B** Fresh chicken RBCs were infected with NDV through both preincubation and coincubation methods, at 3, 6, 12, and 24 h post-infection, the cells were collected, and the copy number of the NP gene was quantified using real-time qPCR. **A** preincubation group; (**B**) coincubation group. **C** RBCs, DF-1, and CEF infected with NDV at an MOI of 1 for 0, 6, 12, 18, and 24 h, the cell culture supernatants were then collected and subjected to a viral titer assay. **D** RBCs, DF-1, and CEF cells were infected with the attenuated NDV strain RFP-LaSota, and viral proliferation was visualized using fluorescence microscopy. (**E** and **F**) RBCs, DF-1, and chicken embryo fibroblasts (CEF) infected with NDV at an MOI of 5 for 2 h at 4 °C, the cells were collected, and the copy number of the *NP* gene was analyzed using real-time qPCR to describe viral adsorption (**E**) and internalization (**F**). Each bar represents the mean ± standard deviation; **P < 0.01.

We further investigated the dynamics of NDV replication in chicken RBCs. In conventional viral infection models, where the medium was replaced 1 hour post-infection (hpi)), NDV nucleoprotein (NP) gene copies were detected in chicken RBCs. However, there was no significant increase in viral copy numbers over time. Interestingly, viral copies declined during the later stages of infection (Figure 2A), which may be linked to RBC damage. This observation was also validated in the coincubation group (Figure 2B).

To clarify these findings, we plotted the proliferation curve of NDV in chicken RBCs, using primary chicken embryo fibroblasts (CEF) and immortalized chicken embryo fibroblasts (DF-1) as controls. Unlike CEF and DF-1, NDV did not proliferate efficiently in chicken RBCs (Figure 2C). To further validate this, we infected RBCs with a red fluorescence-labelled NDV strain (LaSota-RFP). Supporting the above findings, no RFP signal was detected in the chicken RBCs (Figure 2D).

Next, we evaluated the adsorption and internalization stages during the early phase of NDV infection. Compared with CEF and DF-1, chicken RBCs exhibited a stronger ability to adsorb the virus (Figure 2E). However, only a very small number of viruses were successfully internalized into the chicken RBCs (Figure 2F).

In summary, these findings demonstrate that while NDV efficiently adsorbs to chicken RBCs, it fails to replicate effectively within these.

### Chicken RBCs facilitated NDV colonization of organs

**Figure 3:**
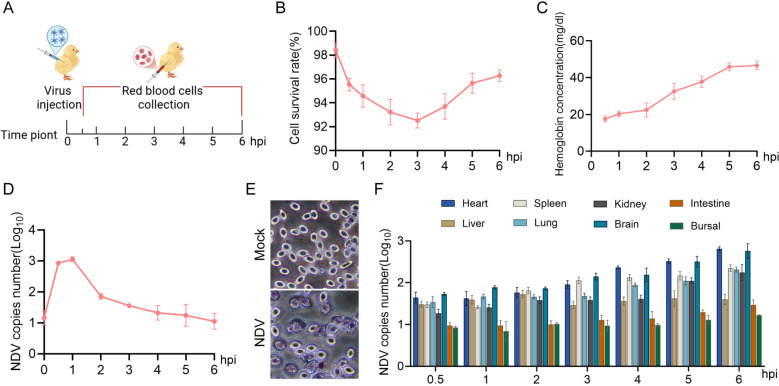
**Chicken RBCs facilitated NDV colonization to organs**. **A** Animal experimental challenge model. **B**–**D** Following viral infection, fresh blood was collected, and red blood cells were isolated at various time points, (**B**) cell viability was assessed; (**C**) plasma free hemoglobin content was measured; (**D**) viral copy numbers were detected using qPCR. **E** After intravenously injecting the virus for 4 h, fresh chicken blood was collected and prepared as blood smears. After Wright’s staining, observations were made under a 40 × microscope. **F** Heart, liver, spleen, lung, kidney, brain, small intestine, and bursa of Fabricius were collected from infected chickens, and the viral copy numbers in different organs were determined.

The strong adsorption capacity of NDV to chicken RBCs suggests a potential mechanism through which NDV utilizes RBC-mediated transport to disseminate throughout the body, colonizing peripheral tissues and organs. To investigate this hypothesis, we established an in vivo NDV challenge model via wing vein injection and collected fresh RBCs through wing vein blood sampling at designated time points post-challenge (Figure 3A).

We first assessed the impact of NDV infection on the viability of red blood cells in chickens using this in vivo model. The results indicated that NDV infection significantly reduced cell viability. Notably, a rebound in viability was observed at 4 h post-injection (Figure 3B), potentially owing to compensatory responses from the host. In addition, plasma hemoglobin levels increased progressively with the advancement of the infection (Figure 3C), indicating that RBCs were damaged and ruptured.

Interestingly, viral copy numbers in RBCs showed a gradual reduction over time (Figure 3D). This decrease may be linked to the release of the virus by RBCs, enabling the establishment of infection in various tissues and organs. Furthermore, blood smear analysis revealed that NDV infection significantly induced marginal shrinkage of chicken RBCs compared with the control group (Figure 3E), suggesting that the intravenous injection of the virus directly caused damage to the RBCs.

We also measured the viral load across different tissues and organs. As expected, the viral load in these organs increased progressively, although the levels varied among them (Figure 3F). These findings demonstrate that RBCs act as transport mediators in the systemic infection process of NDV, facilitating the virus’s colonization of multiple organs.

### NDV triggered apoptosis in chicken RBCs

**Figure 4:**
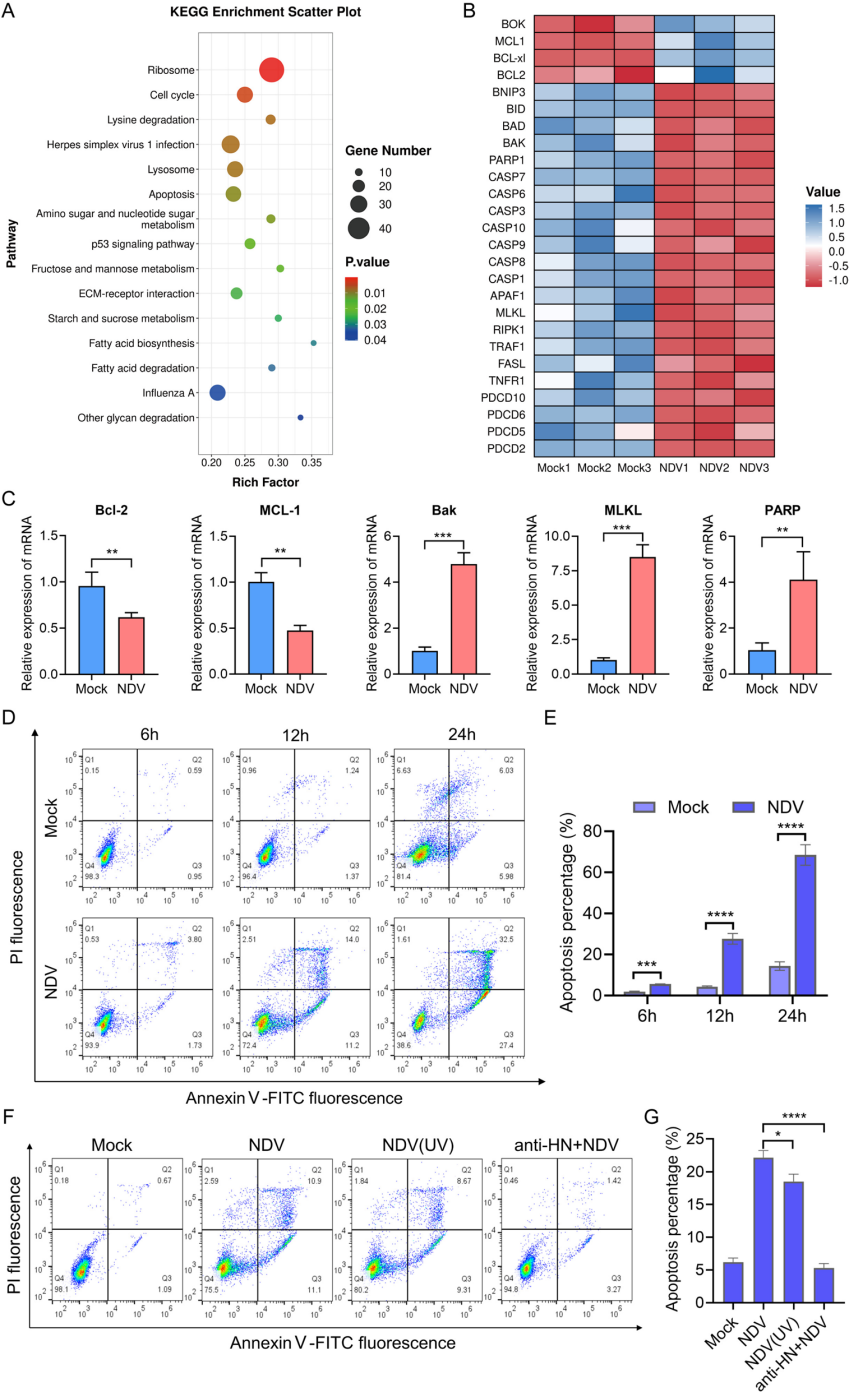
**NDV triggered apoptosis of chicken RBCs. A** Kyoto Encyclopedia of Genes and Genomes (KEGG) enrichment analysis of RBCs differential genes (Mock versus NDV). The horizontal axis depicts the rich factor for each pathway, with the pathway name being indicated on the vertical axis. **B** Differential RBCs apoptosis-related genes content heatmap. Horizontal represents the sample name, vertical represents the metabolite classification. **C** After intravenous injection of NDV 6 h, fresh chicken red blood cells were collected and RNA was extracted. The transcription of Bcl-2, Mcl-1, Bak, MLKL, and PARP genes was analyzed using real-time qPCR. **D** Apoptosis was evaluated using flow cytometry at 6, 12, and 24 h post-infection with MOI of 1 for NDV or a mock infection. **E** The apoptosis rates of cells at different times of infection. Fresh RBCs were infected for 12 h with either inactivated viruses or viruses preincubated with HN antibody. Apoptosis was then analyzed by flow cytometry (**E**) and subsequently quantified (**F**).

To investigate how NDV causes damage to RBCs, we performed transcriptomic profiling on NDV-infected chicken RBCs. Our KEGG enrichment analysis of differentially expressed genes revealed significant enrichment of cell cycle regulation and apoptotic pathways (Figure 4A). Further examination of apoptosis-related genes indicated that NDV infection resulted in the downregulation of antiapoptotic genes from the Bcl-2 protein family in chicken RBCs, while proapoptotic genes were upregulated (Figure 4B). In addition, genes from the Caspase protein family that play roles in cell death regulation, as well as genes associated with necrosis and programmed cell death, were also upregulated (Figure 4B).

To validate our transcriptomic findings, we analyzed the transcriptional levels of several antiapoptotic and proapoptotic genes. Consistent with the transcriptomic data, the levels of anti-apoptotic genes *Bcl-2* and *MCL-1* were downregulated, whereas the proapoptotic gene *Bax* was upregulated. Moreover, the necroptotic effector molecule *MLKL* and the apoptotic effector molecule *PARP* were also found to be upregulated (Figure 4C). These transcriptional shifts suggest that NDV promotes erythrocyte death, which was further corroborated by flow cytometry showing an increase in apoptosis over time following infection (Figures 4D, E).

Interestingly, while NDV usually utilizes host apoptosis to enhance its replication [33], RBCs led to cell death without promoting viral proliferation. To demonstrate that NDV induces apoptosis in chicken RBCs independently of viral replication, we exposed the cells to replication-incompetent, inactivated NDV, which still retained the capacity to induce apoptosis (Figures 4F, G). Importantly, when NDV was preincubated with an HN protein antibody to inhibit viral attachment to RBCs, it effectively blocked NDV-induced red blood cell death (Figures 4F, G). This establishes that NDV initiates apoptosis in chicken RBCs primarily through surface attachment, not replication.

## Discussion

This study uncovers a previously overlooked mechanism by which NDV interacts with chicken RBCs, providing important insights into its systemic pathogenesis. By combining in vitro and in vivo models, we demonstrate that NDV directly damages RBCs in a manner that does not rely on replication. The virus induces apoptosis through surface attachment and uses RBCs as temporary carriers to spread and establish itself in host organs. These findings enhance our understanding of NDV’s pathogenic process and emphasize the dual role of RBCs as both targets for the virus and vehicles for its dissemination.

A key finding of this study is that NDV directly damages chicken RBCs, leading to the release of hemoglobin. This damage is both dose- and time-dependent, as evidenced by decreased cell viability and increased levels of extracellular hemoglobin. The release of hemoglobin may worsen tissue damage by promoting oxidative stress and inflammation, which aligns with the clinical signs of hemorrhages seen in NDV-infected birds.

Free hemoglobin acts as a potent pro-oxidant, facilitating Fenton reactions that produce reactive oxygen species (ROS). These ROS can further injure endothelial cells and intensify inflammation [34, 35]. This mechanism may explain the characteristic pinpoint hemorrhages observed in NDV-infected birds, where oxidative stress from hemoglobin and the cytopathic effects of the virus work together to disrupt vascular integrity.

In addition, the breakdown products of hemoglobin, such as heme and iron, can impact immune responses. Heme activates Toll-like receptor 4 (TLR4), leading to the production of proinflammatory cytokines, while excess iron increases the risk of bacterial coinfections [36].

The transient rebound in RBC viability observed at 4 h post-infection likely indicates compensatory mechanisms at play, such as the splenic removal of damaged RBCs and accelerated erythropoiesis. However, the sustained presence of hemoglobin in the bloodstream suggests these protective defenses are eventually overwhelmed, creating a vicious cycle of RBC damage, oxidative stress, and tissue injury.

Although RBCs do not possess the machinery necessary for NDV replication, our findings emphasize their crucial role as transient viral carriers during systemic dissemination. The impressive ability of RBCs to adsorb NDV likely serves two purposes: it not only facilitates the viral hitchhiking process but may also protect circulating virions from immune surveillance. This protection can extend the lifespan of the virus in circulation and enhance its targeting of specific organs. This mechanism resembles strategies used by the human immunodeficiency virus (HIV), which utilizes RBCs as decoys to evade immune detection while traveling to lymphoid tissues [37].

Over time, we observed a decline in the viral copies associated with RBCs, accompanied by a rise in viral loads in various organs. This suggests a “shedding” mechanism, where virions bound to RBCs detach or are released when RBCs rupture, allowing NDV to colonize tissues and organs. Interestingly, different organs show varying capabilities for viral colonization. For instance, the viral load in the heart and brain increases at a faster rate compared with the bursa of Fabricius, which experiences a slower rise. This disparity may be related to the extent of contact between blood and tissue in different organs, emphasizing the role of RBCs in viral transport.

Further research could quantify the dynamics of viral shedding from RBCs and explore the process of RBC-mediated viral delivery to target organs through real-time tracking of fluorescence-labelled NDV-RBC complexes. Notably, the RBC-mediated transport mechanism may provide evolutionary advantages to NDV. By using RBCs as mobile reservoirs, the virus can circumvent the limitations faced by free virions in plasma, such as a short half-life and susceptibility to extracellular defenses. This strategy may explain the rapid systemic spread of NDV in avian hosts.

It is important to note that NDV cannot replicate in red blood cells. However, this limitation does not stop NDV from triggering apoptosis in chicken red blood cells. Viral attachment, mediated by the HN glycoprotein, seems to be sufficient to initiate apoptotic signalling within these cells. This response is quite different from the cell death induced by NDV in other types of host cells, where apoptosis is typically viewed as a mechanism to facilitate the release of progeny viruses [33].

We hypothesize that apoptosis in red blood cells may result from interactions involving viral surface proteins, likely through HN-mediated binding to sialic acid receptors. Such interactions could inadvertently activate stress response pathways in red blood cells, akin to the “bystander apoptosis” observed in immune cells during viral infections [38, 39]. Further research into the red blood cell surface proteome and its interactions with the HN protein could help clarify this mechanism.

In summary, our findings indicate that chicken red blood cells play an active role in the pathogenesis of NDV. These cells facilitate the spread of the virus through adsorption, induce apoptosis through surface interactions, and exacerbate tissue damage by releasing hemoglobin. Together, these mechanisms enhance the virulence of Newcastle disease and provide a framework for understanding the role of red blood cells in viral infections. By elucidating these pathways, this study offers a more comprehensive understanding of the systemic impact of NDV infection. It highlights the critical role that red blood cells play in viral pathogenesis and provides a valuable model for studying strategies to inhibit viral spread and reduce tissue and organ damage in infectious viral diseases.

## Data Availability

The authors confirm that the data supporting the findings of this study are available within the article.
